# Supplementary material for: Evaluation of Healthcare Students’ Knowledge on Antibiotic Use, Antimicrobial Resistance and Antimicrobial Stewardship Programs and Associated Factors in a Tertiary University in Ghana: Findings and Implications
Source: Antibiotics (Basel). 2022 Nov 22;11(12):1679. doi: 10.3390/antibiotics11121679 (PMC9774439; doi:10.3390/antibiotics11121679)
Supplement: Supplementary file 1 [file antibiotics-11-01679-s001.zip › antibiotics-2022229-supplementary.pdf]

antibiotics

Supplementary Material - Table S1 - QUESTIONNAIRE

SECTION A: PERSONAL INFORMATION

1. Age _______________

2. Gender: Male ______ Female ________

3. Please indicate your Course of study

Doctor of Pharmacy	
Medicine	
Nursing	
Physician Assistantship	

4. Please indicate your year of study

Level 400	
Level 500	

5. Indicate if you have a relative/ a close friend working in health-related fields
   a. Yes, I have b. No, I don't have

6. Indicate if you were exposed to antibiotic training prior to entering the university
   a. Yes b. No

SECTION B: KNOWLEDGE BASED QUESTIONS

Indicate whether or not you are familiar with the following terms;

7. Antibiotic resistance: YES _______ NO ________

8. Antibiotic Stewardship Programs:
   YES _____ NO _______-

ANTIBIOTIC USE

Please Tick in the correct column from options 'Agree', 'Disagree', and 'do not know.'

	Item	Agree	Disagree	Do not know	Correct response
9	Antibiotics are useful for the treatment of viral infections.				Disagree
10	Patients can stop taking antibiotics when the symptomsare improving.				Disagree

11	There is nothing wrong with keeping left-over antibiotic course for the next time treatment of the same type of infection.				Disagree
12	Antibiotics can cause allergic reactions.				Agree
13	Antibiotics can always given as preventive measures to fight against future infections without prescription.				Disagree
14	Common Cold and Sore throat if treated with antibiotics will make patients recover more quickly.				Disagree
15	It's okay to use antibiotics that were given to a friend or family member, as long as they were used to treat the same illness.				Disagree

ANTIBIOTIC RESISTANCE - Knowledge on Antibiotic Resistance and factors that contribute to antibiotic resistance.

NB: Please Tick in the correct column from options 'Agree', 'disagree', and 'do not know.'

	Items	Agree	Disagree	Do not know	Correct response
16	Prescribing broad-spectrum antibiotics increases antibiotic resistance				agree
17	Poor infection control practices by health professionals can cause the spread of antibiotic resistance.				agree
18	Antibiotic resistance is an issue in other countries but not in Ghana.				disagree
19	The use of antibiotics in livestock production and agriculture contributes to antibiotic resistance.				agree
20	Lack of enforcement regulation sometimes permits antibiotics to be purchased without a prescription from pharmacies.				agree
21	Bacteria acquire efflux pumps that extrude the antibacterial agent from the cell before it can reach its target site and exert its effect.				agree
22	Antibiotic resistance occurs when your body becomes resistant to antibiotics.				disagree
23	Beta-lactamase is an enzyme produced by bacteria that can break down aminoglycosides				disagree
24	Antibiotic resistance is only a problem for people who take antibiotics regularly.				disagree

25	There is no resistance for Streptococcus pyogenes bacteria.				agree
26	Inadequate duration of therapy and doses contributes to Antibiotic resistance leading to poor treatment outcomes.				Agree
27	Antibiotic resistance will be a greater clinical problem in the future than it is today.				agree

ANTIMICROBIAL STEWARDSHIP PROGRAM (ASP) – Knowledge on ASP concepts and practices

Please Tick in the correct column from options 'Agree', 'Disagree', and 'Do not know'

	ITEMS	Agree	disagree	Do not know	Correct responses
28	ASP is a phenomenon for which a bacterium gains resistance to an antibiotic				disagree
29	ASP improve patient care.				agree
30	Prescribing physicians are the only professionals who need to understand antimicrobial stewardship.				disagree
31	Improved healthcare hygiene helps to control antibiotic resistance				agree
32	An optimum knowledge of ASP will be important to you in your career.				agree
33	Antibiotic resistance can be minimized by using broad-spectrum therapy after identification and susceptibility testing of infectious bacteria.				disagree
34	Improving techniques for bacterial diagnostics is an ASP practice that will allow combatting of resistant bacteria.				agree
35	Formal teaching on proper usage of antibiotics among healthcare students is an ASP intervention that may minimize the phenomena of antibiotic resistance.				Agree
